# Glucoregulatory disruption in male mice offspring induced by maternal transfer of endocrine disrupting brominated flame retardants in DE-71

**DOI:** 10.3389/fendo.2023.1049708

**Published:** 2023-03-17

**Authors:** Elena V. Kozlova, Bhuvaneswari D. Chinthirla, Anthony E. Bishay, Pedro A. Pérez, Maximillian E. Denys, Julia M. Krum, Nicholas V. DiPatrizio, Margarita C. Currás-Collazo

**Affiliations:** ^1^ Department of Molecular, Cell and Systems Biology, University of California Riverside, Riverside, CA, United States; ^2^ Neuroscience Graduate Program, University of California Riverside, Riverside, CA, United States; ^3^ Division of Biomedical Sciences, School of Medicine, University of California Riverside, Riverside, CA, United States

**Keywords:** metabolic syndrome, diabetes, polybrominated diphenyl ethers (PBDEs), metabolic reprogramming, epinephrine, brown adipose tissue, persistent organic pollutants (POPs), metabolic disrupting chemicals

## Abstract

**Introduction:**

Polybrominated diphenyl ethers (PBDEs) are commercially used flame retardants that bioaccumulate in human tissues, including breast milk. PBDEs produce endocrine and metabolic disruption in experimental animals and have been associated with diabetes and metabolic syndrome (MetS) in humans, however, their sex-specific diabetogenic effects are not completely understood. Our past works show glucolipid dysregulation resulting from perinatal exposure to the commercial penta-mixture of PBDEs, DE-71, in C57BL/6 female mice.

**Methods:**

As a comparison, in the current study, the effects of DE-71 on glucose homeostasis in male offspring was examined. C57BL/6N dams were exposed to DE-71 at 0.1 mg/kg/d (L-DE-71), 0.4 mg/kg/d (H-DE-71), or received corn oil vehicle (VEH/CON) for a total of 10 wks, including gestation and lactation and their male offspring were examined in adulthood.

**Results:**

Compared to VEH/CON, DE-71 exposure produced hypoglycemia after a 11 h fast (H-DE-71). An increased fast duration from 9 to 11 h resulted in lower blood glucose in both DE-71 exposure groups. *In vivo* glucose challenge showed marked glucose intolerance (H-DE-71) and incomplete clearance (L- and H-DE-71). Moreover, L-DE-71-exposed mice showed altered glucose responses to exogenous insulin, including incomplete glucose clearance and/or utilization. In addition, L-DE-71 produced elevated levels of plasma glucagon and the incretin, active glucagon-like peptide-1 (7-36) amide (GLP-1) but no changes were detected in insulin. These alterations, which represent criteria used clinically to diagnose diabetes in humans, were accompanied with reduced hepatic glutamate dehydrogenase enzymatic activity, elevated adrenal epinephrine and decreased thermogenic brown adipose tissue (BAT) mass, indicating involvement of several organ system targets of PBDEs. Liver levels of several endocannabinoid species were not altered.

**Discussion:**

Our findings demonstrate that chronic, low-level exposure to PBDEs in dams can dysregulate glucose homeostasis and glucoregulatory hormones in their male offspring. Previous findings using female siblings show altered glucose homeostasis that aligned with a contrasting diabetogenic phenotype, while their mothers displayed more subtle glucoregulatory alterations, suggesting that developing organisms are more susceptible to DE-71. We summarize the results of the current work, generated in males, considering previous findings in females. Collectively, these findings offer a comprehensive account of differential effects of environmentally relevant PBDEs on glucose homeostasis and glucoregulatory endocrine dysregulation of developmentally exposed male and female mice.

## Introduction

The epidemic rise in obesity, type 2 diabetes (T2D), liver lipid disorders and metabolic syndrome (MetS) cannot solely be attributed to genetic background and lifestyle changes. Considerable evidence points to a potential contribution of endocrine- (EDCs) and metabolism-disrupting chemicals (MDCs) to the rapid increase in the incidence of these metabolic diseases (1). One class of EDCs/MDCs are polybrominated diphenyl ethers (PBDEs), anthropogenic persistent organic pollutants (POPs), that have been widely used as flame retardants in commercial products such as furniture, carpets, automobiles, building materials and electronics since the 1970’s (2). PBDEs are lipophilic additives, which are not chemically bound and can be readily released into the environment and accumulate in biota. PBDEs form 209 theoretical congeners, brominated diphenyl ethers (BDEs), depending on the number of bromine substitutions on their biphenyl backbone, with tetra-, penta- and hexa-substituted PBDEs being most commonly found in humans. Research in experimental animals and epidemiological studies has revealed that PBDEs impart toxicological actions on reproduction (3, 4), neurodevelopment (5) and thyroid homeostasis (6). While these findings have led to a ban on the production and usage of penta- and octa-BDEs by the European Union in 2004 and to the voluntary phase out in the US starting in 2005, biomonitoring data indicates that PBDEs are still found in significant amounts in the placenta (7), fetal blood (8) and breast milk (9). While deca-BDEs have been only restricted recently in 2022 (US EPA 2022), PBDE emission from in-use and waste stocks, due to inadvertent recycling, is predicted to continue until 2050 (10). The deca-brominated congener, BDE-209, impairs liver gene markers of glucose homeostasis (11) and may be debrominated *in vivo* to more harmful penta-, hepta-, octa- and nona-brominated congeners after penetration (12–14). Therefore, continued emissions from and the disposal of PBDE-containing products that are still in use is an ongoing challenge and the metabolic disruptive effects of PBDEs warrants further study.

The T2D epidemic has become a serious global health issue, affecting 415 million people world-wide (15) and poses a significant economic burden on individuals and society (16). A large body of evidence from human (17–23), and animal research (24–29), supports the idea that brominated flame retardants (BFRs), such as PBDEs, may be risk factors for T2D and MetS. A commonality in many of these studies is that diabetes and/or MetS are positively associated with body burdens of the PBDE congeners BDE-28, -47, or -153, all commonly found in humans and biota. These are found in DE-71, a commercial mixture of PBDEs with environmental relevance (30). Our past work shows that these and other DE-71 congeners, at the human-relevant doses used in the current study, can be transferred perinatally *via* exposed dams and accumulate in male and female offspring liver and brain ((31, 32), and unpublished observations). DE-71 may lead to permanent alterations in metabolic status in offspring, especially if exposure is administered during a period of high biological plasticity in offspring such as gestation and lactation. Indeed, we have shown that female offspring exposed to 0.1 mg/kg/d DE-71 perinatally, exhibit alterations in clinically relevant biomarkers of T2D - elevated fasting blood glucose, glucose intolerance, insulin resistance, altered plasma levels of glucoregulatory hormones and liver endocannabinoid tone, an emerging biomarker of energy balance. Further, we have found that PBDEs produce sex-dependent changes in hypothalamic peptidergic circuits that control food intake and energy metabolism (29).

The interplay between the key endocrine regulators of glucose homeostasis, glucagon, insulin, and glucagon-like-peptide (GLP-1) is informative in understanding the diabetic phenotype of T2D. The incretin GLP-1 is secreted by enteroendocrine L-cells and acts on pancreatic β-cells to promote postprandial insulin secretion. In T2D, this insulinotropic effect is either impaired or absent leading to hyperglycemia (33). Activation of central and peripheral GLP-1 receptors also suppresses the secretion of glucagon, contributing to normoglycemia. However, in T2D, glucagon levels are elevated, potentially due to a lack of inhibitory tone of insulin on glucagon (34). Our past work provides evidence for endocrine disruption of these key glucoregulatory hormones by DE-71, namely downregulation of insulin in L-DE-71-exposed diabetogenic female offspring and upregulation of GLP-1 in dams exposed in adulthood (31). EDCs and MDCs, including PBDEs, have been shown to alter insulin levels (19, 35), and increase the risk of gestational diabetes (17). However, little is known about potential sex differences in the EDC effects of PBDEs on metabolic hormones and there is scant information about sex differences in the endocrine pathophysiology of T2D, although several hormones regulating glucose control can vary by sex and body type (36, 37). This prompted us to study the EDC and MDC effects of early-life exposure to PBDEs on male offspring. We used an integrative physiological approach that examines pertinent organ systems.

Having observed sexually dimorphic risk and susceptibility of perinatally exposed females to MetS (28, 29), the purpose of the current study was to comprehensively examine the effect of DE-71 on glucose homeostasis in male offspring. Using a mouse model of chronic, low-dose maternal transfer of environmentally relevant PBDE congeners, we tested the hypothesis that exposure to DE-71 produces a diabetogenic phenotype in male offspring. Results indicate that exposure to DE-71, alters clinically relevant diabetic biomarkers, namely, fasting blood glucose, glucose tolerance, insulin sensitivity, plasma levels of glucoregulatory hormones and other hepatic, adipose and adrenal effects. While no direct comparisons were made between the previous results in females and current results in males, the pattern of glucoregulatory disruption in exposed males has different characteristics than that observed in exposed females. Taken together, the current study and our past work elucidate a comprehensive profile of clinically relevant and persistent metabolic disruption induced by PBDEs and raises concern for the progeny of directly exposed mothers.

## Methods

### Animals

C57BL/6N mice were obtained from both Charles River Laboratories (Raleigh, NC) and Taconic Biosciences (Germantown, NY). Mice were group housed 2-4 per cage and maintained in a non-specific pathogen free vivarium on a 12 h light/dark cycle at an ambient temperature of 20.6-23.9 °C and relative humidity of 20-70%. Mice were provided rodent chow (Laboratory Rodent Diet 5001; LabDiet, USA) and municipal tap water *ad libitum* in glass water bottles. Procedures on the care and treatment of animals were performed in compliance with the National Institutes of Health *Guide for the Care and Use of Laboratory Animals* and approved by the University of California, Riverside, Institutional Animal Care and Use Committee (AUP#20170026 and 20200018).

### DE-71 dosing solutions

Dosing solutions were prepared as described previously (31, 32). In brief, technical grade pentabromodiphenyl ether mixture (DE-71; Lot no. 1550OI18A; CAS 32534-81-9), was obtained from Great Lakes Chemical Corporation (West Lafayette, IN). DE-71 dosing solutions were prepared in corn oil (Mazola) to yield two low doses: 0.1 (L-DE-71) and 0.4 (H-DE-71) mg/kg bw per day (2 uL/g body weight). Vehicle control solution (VEH/CON) contained corn oil without the addition of DE-71. PBDE doses were chosen to yield desired body burdens that are relevant to humans. Our recent data indicate that transfer and accumulation of PBDE congeners is similar in male and female tissues (32 and unpublished observations). These findings detail the levels and composition of BDEs analyzed in DE-71-dosed mice.

### DE-71 exposure

**Figure 1:**
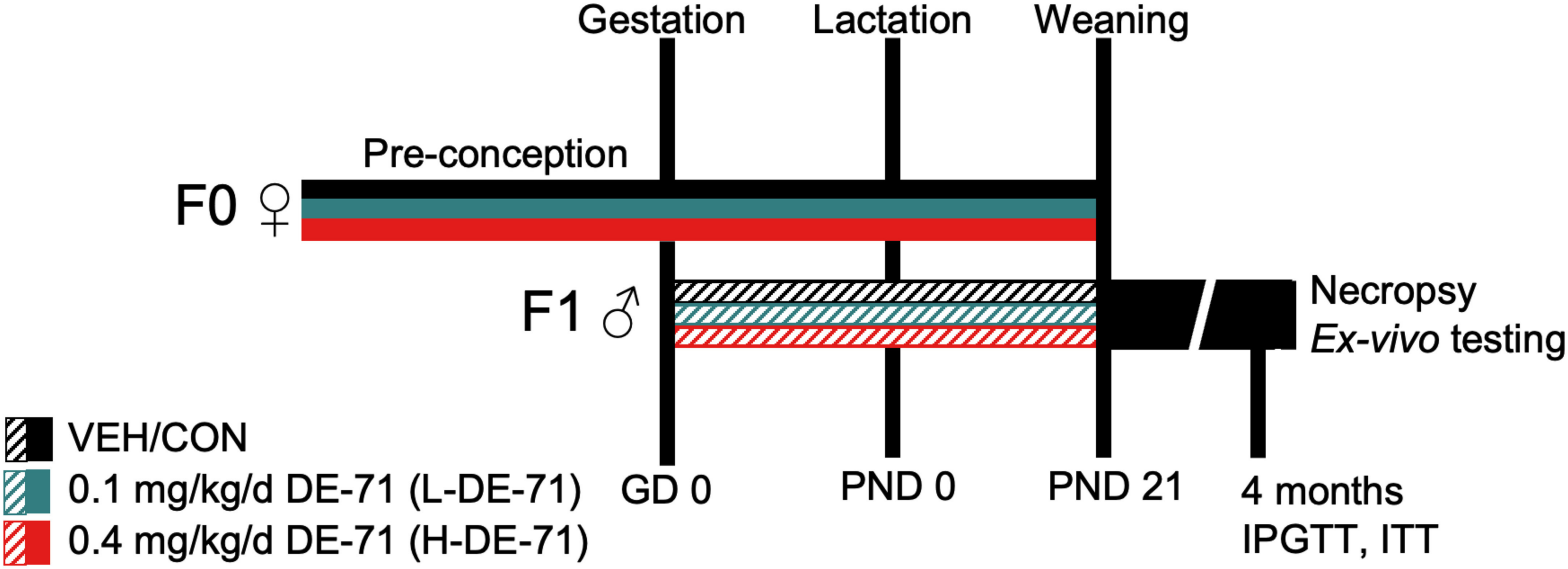
Diagram depicting the dosing and testing paradigm used for perinatal exposure to DE-71. Direct exposure of dams to DE-71 (F0♀, solid shading), began ~4 weeks pre-conception and continued daily through ~3 weeks of gestation and 3 weeks of lactation until pup weaning at PND 21. Indirect exposure to DE-71 in male offspring (F1♂, hatched shading) occurred during the prenatal (GD 0- 20) and postnatal periods (PND0-PND21) *via* lactation. Offspring received 39 days of DE-71 exposure during the prenatal period except on PND 0 and 1. Metabolic endpoints (fasting glycemia, GTT, ITT) were examined in Cohort 1 offspring at ~4 months of age. Another cohort (Cohort 2) of animals was raised for ex vivo analyses. At necropsy (~4 mo of age) Cohort 2 body and organ weights were recorded and blood and organ tissues collected for further analysis: plasma hormones, UPLC/MS/MS detection of endocannabinoids, adrenal epinephrine and liver GDH enzymatic activity. GD, gestational day; PND, postnatal day; GDH, glutamate dehydrogenase.

Perinatal PBDE exposure *via* the dam was accomplished as described previously (31). In brief, female mice (PND 30-60) were introduced to cornflakes daily for 1 week. Dams were randomly assigned to one of three exposure groups: corn oil vehicle control (VEH/CON), 0.1 (L-DE-71) or 0.4 mg/kg bw per day DE-71 (H-DE-71) (**Figure 1**). A 10-week dosing regimen, chosen to model human-relevant chronic, low-level exposure (18, 20, 38), included ~4 weeks of pre-conception, plus gestation (3 weeks) and lactation (3 weeks). Offspring were weaned after the lactation period at PND 21 and housed in same-sex groups. Dams were fed oral treats (Kellogg’s Corn Flakes) infused with dosing solution (2 uL/g bw) daily, except on PND 0 and 1. Each dam was exposed to DE-71 daily for an average of 70-80 d and offspring were perinatally exposed for 39 d *via* maternal blood and milk. Consumption was visually confirmed, and offspring were never allowed to ingest cornflakes. DE-71 exposure does not significantly affect maternal food intake, gestational weight gain, litter size or sex ratio (31). Male offspring were used *in vivo* for physiological testing (Cohort 1) and *ex vivo* analysis of hepatic, adipose, adrenal, and endocrine parameters (Cohort 2). The DE-71 exposure and testing paradigm is shown in **Figure 1**.

### Glucose tolerance test and insulin tolerance test

Mice were fasted overnight (ON) for 11h and then injected with glucose (2.0 g/kg bw, i.p.). Glucose was measured directly from tail blood (~1 uL) at time 0, 15, 30, 60, and 120 min post-glucose challenge. A calibrated glucometer and test strips (OneTouch Ultra 2, LifeScan Inc.) were used to measure plasma glucose concentrations. Seven days following IPGTT, an insulin tolerance test (ITT) was performed with Humulin R (Eli Lilly) bolus (0.25 U/kg bw, i.p.) on mice fasted ON for 9h. Tail blood was collected, and glucose was sampled in the same manner as IPGTT (t=0, 15, 30, 45, 60, 90 and 120 min). For area calculations, the area under (AUC) or above the glycemia curve (inverse AUC) from 0-120 min post injection was used. The percent blood glucose reduction rate after insulin administration, K_ITT_, was calculated using the formula (0.693 x 100) x t_1/2_^-1^ to determine *in vivo* insulin sensitivity from 0-30 min post insulin injection. Fasting blood glucose was determined from baseline values (t=0) obtained in IPGTT (11h) and ITT (9h). GTT and ITT sample sizes were different because ITT effect size was expected to be less (as estimated by our previous work (31)) and, therefore, more samples were needed in ITT, i.e., 14 additional samples, vs GTT.

### Necropsy

During sacrifice, under terminal isoflurane anesthesia, cardiac blood (0.3-1 mL) was collected and centrifuged at 16,000 x g for 20 min at 4 °C. After the addition of a cocktail of protease inhibitors and EDTA, the plasma samples were stored at -80°C until further use. The following organs were excised and weighed: liver, pancreas, spleen, adrenal glands and interscapular brown adipose tissue (BAT). Plasma, liver and adrenal samples were snap-frozen over dry ice and stored at -80 °C for later analysis of plasma hormones, adrenal epinephrine, liver endocannabinoids and enzymatic activity.

### Enzyme-linked immunosorbent assays (EIAs)

Plasma collected *via* cardiac puncture (*ad libitum* fed state) was analyzed for metabolic hormones using commercially available kits according to manufacturer’s instructions. Plasma insulin was measured using commercial ELISA kits (ALPCO, Salem, NH, Cat.# 80-INSMS-E01 and Mercodia, Uppsala, Sweden Cat.# 10-1249-01 and 10-1247-01) as previously described (31). The Mercodia assays had a sensitivity of 0.15 mU/L or 1 mU/L in a standard range of 0.15-20 mU/L or 3-200 mU/L and inter- and intra-assay coefficient of variance (CV) of 4.9% and 3.4%, respectively. The ALPCO insulin ELISA had a sensitivity of 0.019 ng/mL in a standard range of 0.025-1.25 ng/mL and inter- and intra-assay CV of 5.7% and 4.5%, respectively. The active glucagon-like peptide-1 (7–36) Amide (GLP-1) assay (Cat.# 80-GLP1A-CH01, ALPCO) had an analytical sensitivity of 0.15 pM in a standard range of 0.45-152 pM and inter- and intra-assay CV of 11.6 and 9.5%, respectively. Glucagon was measured by chemiluminescence using a proprietary ELISA kit (ALPCO) undergoing beta launch development (now Cat.# 48-GLUHU-E01). This assay had a sensitivity of 41 pg/mL in a dynamic range of 41-10,000 pg/mL and inter- and intra-assay CV of 9.8% and 7.6%, respectively.

### Measurement of hepatic endocannabinoids using ultra-performance liquid chromatography-tandem mass spectrometry

Hepatic lipids were extracted using a modified Folch method (31). Samples of flash-frozen liver tissue were weighed (10-20 mg) and homogenized in 1 mL of methanol solution containing 1 pmol d_4_-arachidonoylethanolamide (internal standard for quantitation of arachidonoylethanolamide), 10 pmol d_4_-oleoylethanolamide (internal standard for quantitation of oleoylethanolamide and docosahexaenoylethanolamide), and 500 pmol d_5_-2-arachidonoyl-*sn*-glycerol (internal standard for quantitation of 2-arachidonoyl-*sn*-glycerol and 2-docosahexaenoyl-*sn*-glycerol). Following homogenization of samples, 2 mL of chloroform and 1 mL of water were added, followed by centrifugation at 2000 x g for 15 min at 4°C. The organic phase was extracted and the pooled lower phases were dried under N_2_ gas followed by resuspension in 0.1 mL methanol:chloroform (9:1). Analysis of endocannabinoids (EC) was performed *via* ultra-performance liquid chromatography coupled to tandem mass spectrometry (UPLC/MS/MS) as previously described (39, 40).

### Glutamate dehydrogenase (GDH) activity

GDH activity in crude liver homogenates was assayed using the tetrazolium salt method with modification for multiwell plates as described previously (41, 42). In brief, 5% liver homogenates (40 uL in 0.25M sucrose) were added to a mixture of 2 μmol/l of iodonitrotetrazolium chloride, 0.1 μmol/l of NAD, 50 μmol/l of sodium glutamate, 100 μmol/l of phosphate buffer (pH 7.4) and distilled water. Samples were run in duplicate. The reaction was allowed to proceed at 37°C for 30 min. The optical density of the resultant formazan product was measured at 545 nm and then converted to concentration using a iodonitrotetrazolium formazan (TCI) standard curve fitted with a linear regression model (42). A bicinchoninic acid assay (Cat.# 23227, ThermoFisher Scientific, Waltham, MA, USA) was used to measure protein content in order to normalize product yield values. GDH activity was expressed as µmol formazan formed per ug protein/h.

### Epinephrine assay

Epinephrine content in adrenal glands was measured using a modification of the trihydroxyindole method as described previously (43). Briefly, 5 mg of adrenal tissue homogenates (in 200 µL of 0.05 N perchloric acid) were centrifuged at 15,000 x g at 0°C for 15 min. Acetic acid (10%, pH 2) was added to the sample supernatant (30 uL), followed by 60 µL of 0.25% K_2_Fe(CN)_6._ The mixture was incubated at 0°C for 20 min and the oxidation reaction was stopped by the addition of 60 µL of a 9 N NaOH solution containing 4 mg/ml ascorbic acid (alkaline ascorbate). Fluorescence emission was determined at 520 nm (excitation wavelength at 420 nm) using a fluorescence plate reader (Promega). Epinephrine concentration (μg/g adrenal wet weight) was converted from the mean fluorescence intensity units of each sample using calibration standards and polynomial curve fitting.

### Statistical analysis

Statistical analyses were performed using GraphPad Prism v.9.4.1. A one-way analysis of variance (ANOVA) was used to test the main effect of one factor. When normality assumption failed, as determined using a Shapiro-Wilk test, a Kruskal-Wallis H ANOVA was used. A Brown-Forsythe ANOVA was used if the group variances were significantly different. Data for fasting glycemia were analyzed by two-way ANOVA for main effects of exposure and fasting duration. ITT and GTT experiments were analyzed by repeated measures two-way or mixed model ANOVA to determine main effects of exposure and time. ANOVA was followed by *post hoc* testing for multiple group comparisons. For parametric ANOVAs, Tukey’s *post hoc* test was used when both sample sizes and variance were equal, and a Dunnett’s T3 test was used when sample size and variance were not equal. For non-parametric ANOVA, a Dunn’s *post hoc* test was performed. For Two-way ANOVA, Tukey’s, Sidak’s and Holm-Sidak’s *post hoc* tests were used. Differences were deemed significant at *p*<0.05. Data are expressed as mean ± standard error of the mean (s.e.m) unless indicated otherwise.

## Results

### Chronic low dose DE-71 exposure has minimal effects on body and organ weights

**Table 1** shows the effects of DE-71 exposure on body weights, select organ weights (absolute weights) and organ-to-body-weight-ratios (relative weights). At necropsy, body weights of male offspring were not different across groups and, therefore, the diabetogenic phenotype of DE-71-exposed male mice is not due to obesity. This is consistent with our previous findings of no difference in lean or fat mass in DE-71-exposed males (29). Absolute liver weight was 9% lower in L-DE-71 relative to VEH/CON (One-way ANOVA: *Exposure effect* F_(2,58)_=9.34, *p*<0.001, Tukey’s *post-hoc* VEH/CON vs L-DE-71, *p*=0.03 and lower in L-DE-71 vs H-DE-71, *p*=0.0003). Relative liver weight was 8% greater in H-DE-71 relative to L-DE-71 (One-way ANOVA: *Exposure effect* F_(2,58)_=2.824, *p*=0.0675, Tukey’s *post-hoc* L-DE-71 vs H-DE-71 *p<*.05). The absolute and relative weights of pancreas and spleen were similar across groups.

**Table 1:** Body and select organ weights in de-71 exposed male mice.

n	VEH/CON	L-DE-71	H-DE-71
	17	28	18
Necropsy body weight	25.7±0.50	24.8±0.71	25.7±0.49
Liver			
Absolute	1.16±0.03	1.06±0.02*	1.21±0.03^^^
Relative	44.8±1.70	43.2±1.05	47.1±1.05^
Pancreas			
Absolute	0.17±0.007	0.17±0.007	0.19±0.008
Relative	6.37±0.26	6.94±0.21	7.27±0.30
Spleen			
Absolute	0.07±0.002	0.07±0.002	0.07±0.003
Relative	2.64±0.10	2.85±0.10	2.80±0.09

Values for body and organ weights (absolute weight) are expressed in grams; organ weight/body weight (relative weight) are expressed as mg organ weight/g body weight. Values are reported as mean ± standard error of the mean (s.e.m.).

*Indicates significantly different from VEH/CON (*p<0.05).

^Indicates significantly different relative to L-DE-71 (^*p*<0.05; ^^^*p*<0.001).

### DE-71 produces fasting hypoglycemia

**Figure 2:**
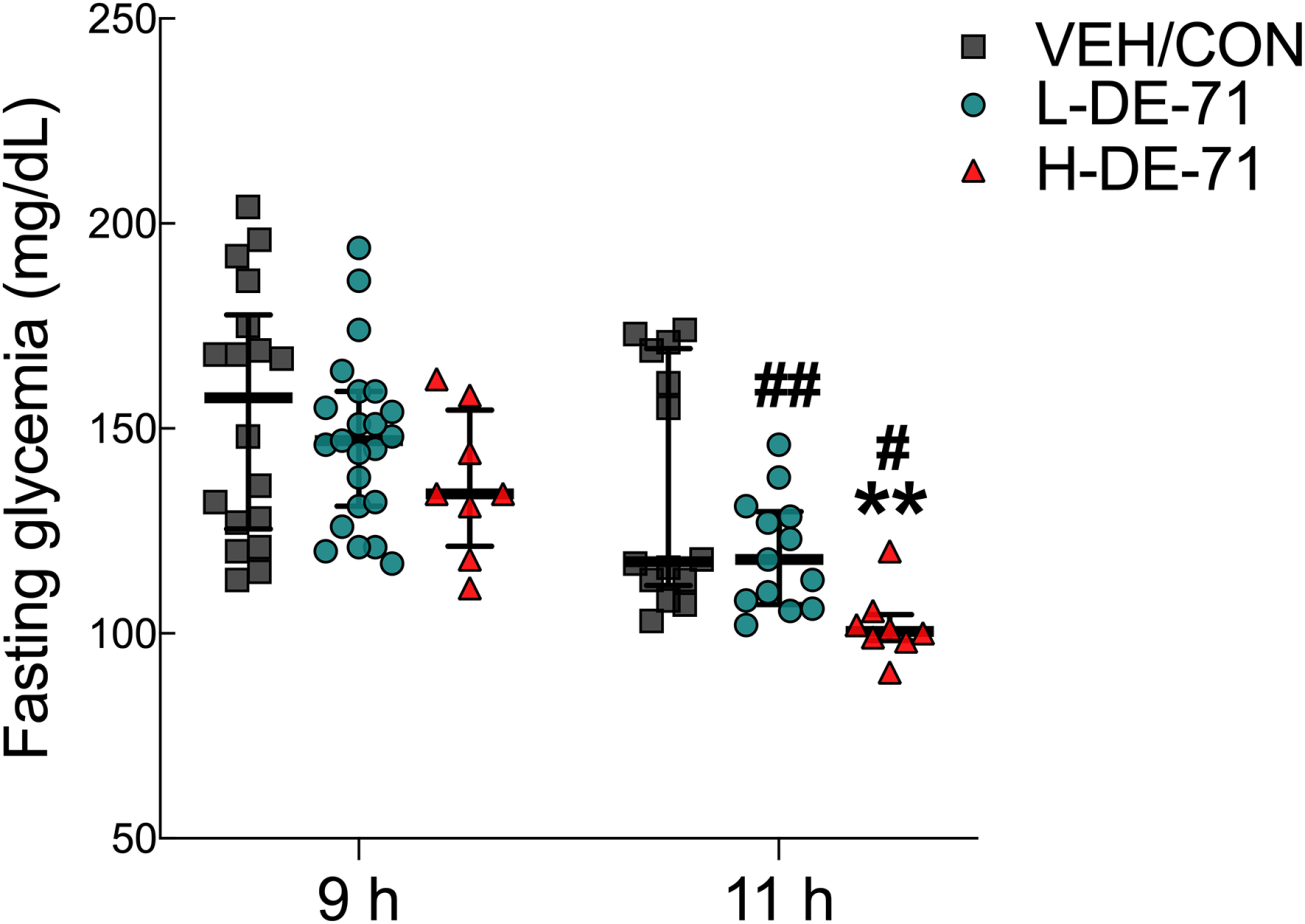
Fasting blood glucose (FBG) in male offspring exposed perinatally to DE-71. FBG was measured after a 9h fast in conjunction with ITT and 11h fast before GTT in male offspring. *indicates significantly different from VEH/CON (**p<0.01). ^#^indicates significant difference vs 9 h fast for corresponding exposure group (^#^ *p<*0.05; ^##^ *p*<0.01). Bars and error bars represent values expressed as median with interquartile range. *n*, 8-23/group.

We examined fasting blood glucose (FBG) after 9 and 11 h fasting using glycemia values from basal time points obtained in ITT and GTT experiments, respectively. **Figure 2** shows that exposure to H-DE-71 significantly lowered (25%) FBG after a 11 h fast relative to VEH/CON (Two-way ANOVA: *Exposure effect* F_(2,78)_= 6.65, *p*<0.01; *Time effect* F_(1,78)_= 24.6, *p*<0.0001; *Exposure* x *Time* F_(2,78)_= 0.76 ns; Sidak’s *post hoc* test, 11 h: VEH/CON vs H-DE-71, *p*<0.01; 9 h vs 11 h, L-DE-71 *p*<0.01, H-DE-71, *p*<0.05 (**Figure 2**). In comparison, this was not observed after a shorter 9 h fast. These results suggest that perinatal exposure to DE-71 lowers fasting glycemia but only after a prolonged fast.

### DE-71 exposure impairs glucose tolerance

**Figure 3:**
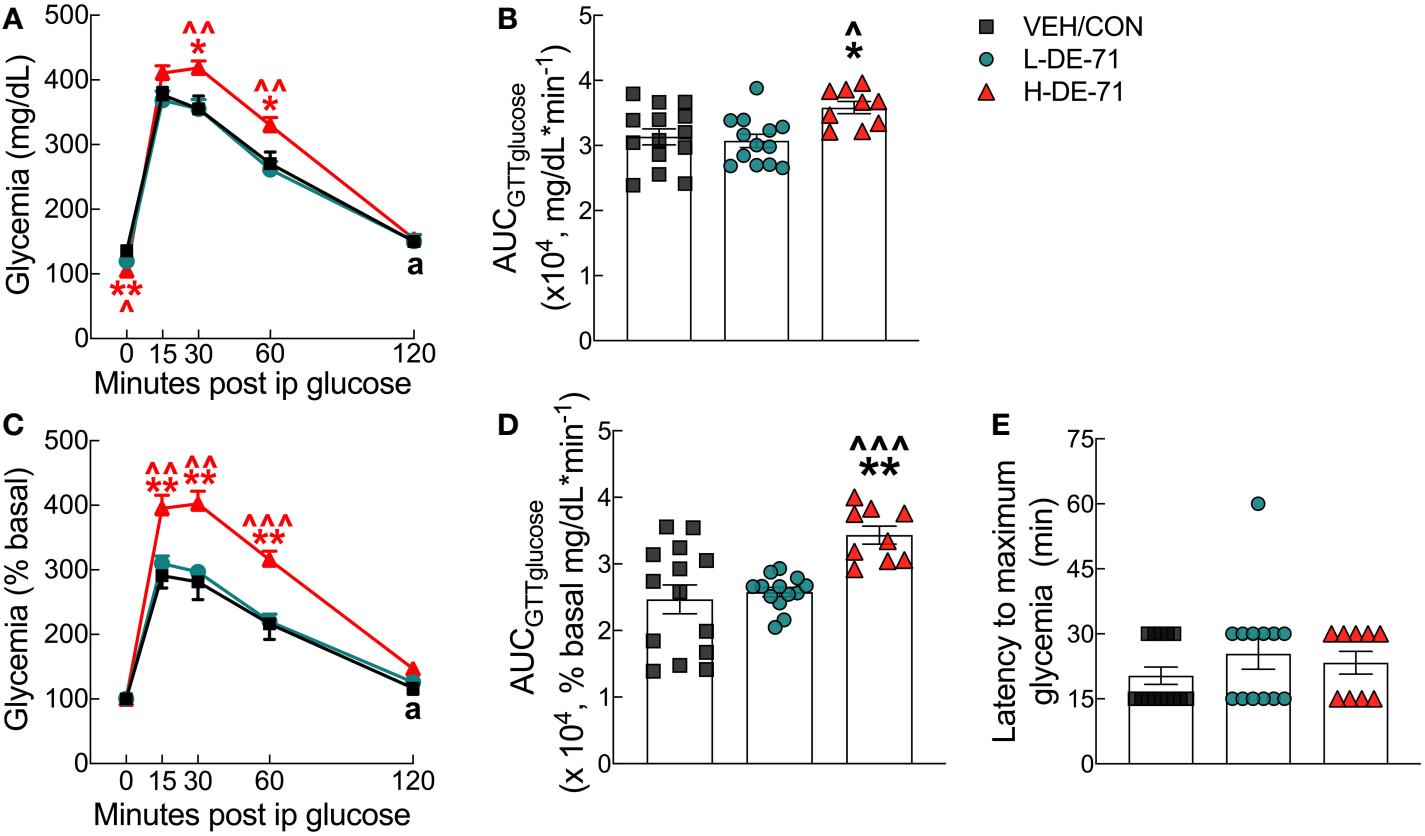
Glucose tolerance profile in male mice offspring receiving perinatal exposure to DE-71. Mice were fasted for 11 h ON and tail blood was sampled for glucose before (t=0 min) and after (t=15, 30, 60 and 120 min) i.p. injection of 2.0 g/kg glucose. **(A)** Absolute blood glucose concentrations taken during IPGTT. **(B)** Mean values for the integrated area under the IPGTT glucose curve using absolute values (AUC_GTTglucose_). **(C)** Blood glucose values taken during IPGTT are plotted vs time as a percent of basal glucose. **(D)** Mean values for the integrated area under the IPGTT glucose curve using percent baseline values (AUC_GTTglucose_). **(E)** Latency to maximum glycemia corresponding to percent basal. Asterisk of specific color indicates significant difference between corresponding group and VEH/CON (*p<0.05, **p<0.01). Carets of specific color indicate significant difference between corresponding group and L-DE-71 (^*p*<0.05, ^^*p*<0.01, ^^^*p*<0.001). The black symbol “a” in panels **(A, C)** indicates the time points at which glycemia is not different from basal (t=0) in the VEH/CON group. Glycemia at all other time points and groups differs from basal. Bars and error bars represent values expressed as mean ± s.e.m. *n*, 9-14/group.

To investigate the effects of DE-71 on glucose tolerance, glycemia was measured during GTT over the 120 min post-injection time course (**Figure 3A**). Blood glucose levels rose rapidly and peaked between 15-30 min of glucose challenge in all groups. Peak glycemia was exaggerated in H-DE-71 males relative to VEH/CON (RM Two-way ANOVA: Exposure effect F_(2,33)_=4.767, *p*<0.05; *Time effect* F_(2.9,96.1)_=330.2, *p*<0.0001; *Time* x *Exposure* F_(8,132)_ =3.112, *p*<0.01) (**Figure 3A**). Mean glycemia values in H-DE-71 were markedly elevated as compared to VEH/CON at *t*=30 (Holm-Sidak’s *post hoc*; *p*<0.05) and 60 min post injection (*p*<0.05) and in L-DE-71 vs H-DE-71 at *t*=30 (*p*<0.01) and 60 min post injection (*p*<0.01).

Since FBG after an 11 h fast was lower in H-DE-71 relative to VEH/CON (t=0, *p*<.01; **Figure 2**), glycemia values are also expressed as percent baseline (**Figure 3C**). After normalizing to baseline (t=0), peak glycemia was again exaggerated in H-DE-71 males relative to VEH/CON (RM Two-way ANOVA: *Time effect* F_(2.26, 74.7)_=278.6, *p*<0.0001; *Exposure effect* F_(2,33)_=9.39 *p*<0.001; *Time* x *Exposure* F_(8,132)_=5.94, *p*<0.0001). Mean glycemia values for H-DE-71 were significantly different from VEH/CON at *t*=15 (Tukey’s *post hoc*; *p*<0.01), 30 (*p*<0.01) and 60 min post injection (*p*<0.01) and from L-DE-71 at *t*=15 (*p*<0.01), *t*=30 (*p*<0.01) and 60 min post injection (*p*<0.001).

The differences in magnitude and duration of glycemia are integrated using the area under the glucose curve, AUC_GTTglucose_, which is abnormally large in H-DE-71 males (Absolute glycemia AUC, One-way ANOVA: *Exposure effect* F_(2,33)_=5.21, *p*<0.05, Tukey’s *post-hoc* VEH/CON vs H-DE-71, *p*<0.029, L-DE-71 vs H-DE-71, *p*<0.013; Percent basal glycemia AUC, Brown-Forsythe ANOVA: *Exposure effect* F_(2,22.2)_=10.1, *p*<0.001, Dunnett’s T3 *post-hoc* VEH/CON vs H-DE-71, *p*<0.004, L-DE-71 vs H-DE-71 *p*<0.0003 (**Figures 3B, D**). The latency to maximum glycemia, likely influenced by the insulin response, was not significantly different across groups (**Figure 3E**). Of note, glycemia was still elevated in both exposure groups (L- and H-DE-71) but not VEH/CON at 120 min post-glucose injection (“a” in **Figures 3A, C**). These comparisons are significant when comparing percent basal glycemia at 120 min vs t=0 (L-DE71 *p*<0.05-.01, H-DE-71 *p*<0.001).

### L-DE-71 exposure produces an abnormal glycemic response to insulin

**Figure 4:**
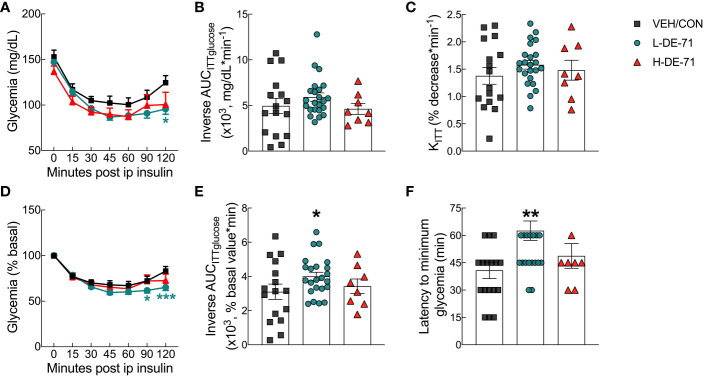
Abnormal glycemia reduction after insulin challenge in male mice offspring exposed perinatally to L-DE-71. **(A)** Absolute blood glucose concentrations were recorded before and at *t*=15, 30, 45, 60, 90 and 120 min post-injection with 0.25 U/kg insulin. **(B)** Tolerance to insulin challenge was analyzed by the inverse integrated area under the ITT glucose curve (AUC_ITTglucose_). **(C)** Rate constant for glucose reduction (K_ITT_) was calculated over the initial slope of ITT glucose response curve from 0-30 min post-injection. **(D)** Glucose values taken during ITT are plotted vs time as a percent of the individual baseline. **(E)** The inverse integrated area (AUC) under the percent basal glucose curve (AUC_ITTglucose_). **(F)** Latency to minimum blood glucose measured over the two-hour time course of ITT glucose response. *indicates significantly different from VEH/CON (*p<0.05, **p<0.01, ***p<0.001). All values are expressed as mean ± s.e.m. *n*, 8-23/group.

The glycemia response to exogenous insulin was examined during ITT experiments. The insulin tolerance curve shows mean glycemia values over the 120 min period following insulin injection (**Figure 4A**). Males exposed to L-DE-71 display greater reduction in glycemia as compared to VEH/CON (RM Two-way ANOVA: *Time effect* F_(3.257,143.3)_=56.2, *p*<0.0001; *Exposure effect* F_(2,44)_=2.40, *ns*; *Time* x *Exposure* F_(12,264)_=1.90, *p*<0.05) (**Figure 4A**). Mean glycemia values for L-DE-71 were significantly different from VEH/CON at *t*=120 min (Tukey’s *post hoc*; *p*<0.05). When glycemia is expressed as a percent of baseline, L-DE-71 exposed mice displayed incomplete recovery or glucose clearance/utilization at 90 and 120 min after insulin injection (RM Two-way ANOVA: *Time effect* F_(6,308)_=33.7, *p*<0.0001; *Exposure effect* F_(2,308)_=8.014, *p*<0.001; *Time* x *Exposure* F_(12,308)_=1.23, *ns*) (**Figure 4D**). Mean glycemia values for L-DE-71 are significantly different from VEH/CON at *t*=90 (Tukey’s *post hoc*; *p*<0.05) and 120 min (*p*<0.001). This is represented as a greater mean latency to reach the minimum insulin-induced hypoglycemia in L-DE-71 relative to VEH/CON (Kruskal-Wallis test: Exposure H(2)=9.07, *p*<0.01; Dunn’s *post hoc* VEH/CON vs L-DE-71, *p*<0.01) (**Figure 4F**).

The inverse area under the glucose response curve (inverse AUC_ITTglucose_) was plotted using absolute (**Figure 4B**) and percent baseline values (**Figure 4E**). The latter showed a significant increase for L-DE-71 relative to VEH/CON (One-way ANOVA: *Exposure effect* F_(2,44)_=2.024, ns, Sidak’s *post-hoc* VEH/CON vs L-DE-71, *p*<0.05) (**Figure 4E**). To measure the early effects of insulin sensitivity, we calculated K_ITTinsulin_ measured over the first 30 min post-injection (**Figure 4C**). There were no differences between groups for this metric, suggesting no differences in early glucose uptake triggered by insulin. Interestingly, male offspring of dams exposed to L-DE-71 during pregnancy show a delayed recovery from insulin challenge.

### Endocrine-disrupting effects of DE-71 exposure on glucoregulatory hormones

**Figure 5:**
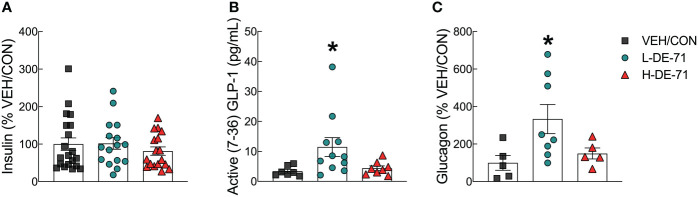
Endocrine-disrupting effects of DE-71 on glucoregulatory hormones in male mice. Blood collected at sacrifice was assayed for plasma levels of **(A)** insulin **(B)** GLP-1 and **(C)** glucagon using specific commercial enzyme-linked immunoassay kits. *indicates significantly different from corresponding VEH/CON (*p<0.05). Bars and error bars represent values expressed as mean ± s.e.m. *n*=16-20/group for insulin, *n*=7-11/group for GLP-1, *n*=5-8/group for glucagon.

We have previously shown disrupted plasma hormones involved in carbohydrate regulation in DE-71 exposed female offspring. In T2D, pancreatic beta cell dysfunction may lead to changes in insulin production (44). In addition, elevated fasting and postprandial plasma glucagon concentrations have also been shown and may contribute to symptomatic hyperglycemia (33, 45). We used ELISA to measure plasma insulin, active GLP-1 (7-36) amide and glucagon in blood collected at necropsy (*ad libitum* fed state) (**Figures 5A–C**). Plasma levels of insulin and glucagon were expressed as percent of control but mean absolute concentrations ranged from 0.90 ± 0.12 to 1.12 ± 0.17 ug/L for insulin, 12.6 ± 4.98 to 41.9 ± 9.78 pg/mL for glucagon in F1 males. For glucagon ELISA, there were no commercially available kits at the time of our studies so we used proprietary kits undergoing beta launch development. Therefore, we went through several trials and samples were expended that did not yield data. Hence the sample size was smaller in **Figure 5C**. With regard to plasma insulin levels, there were no group differences (**Figure 5A**). However, L-DE-71 males showed elevated plasma GLP-1 and glucagon relative to VEH/CON (GLP-1, One-way ANOVA: *Exposure effect* F_(2,23)_=3.684, *p*=0.0409; Dunnet’s *post-hoc* VEH/CON vs L-DE-71 *p*=0.0473; Glucagon, One-way ANOVA: *Exposure effect* F_(2,11.3)_=5.61, *p*=0.02; Dunnett’s T3 *post-hoc* VEH/CON vs L-DE-71 *p*=0.0449) (**Figures 5B, C**). In summary, L-DE-71 showed the most susceptibility to the endocrine-disrupting effects of PBDEs.

### Upregulated adrenal epinephrine content and reduced BAT after DE-71 exposure

**Figure 6:**
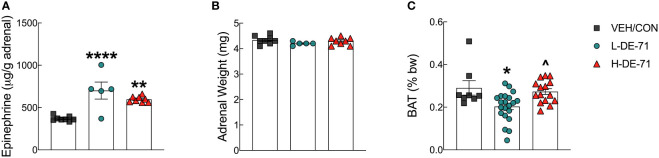
Effects of DE-71 on adrenal epinephrine content and thermogenic brown adipose tissue mass. **(A)** Epinephrine content measured in adrenal glands harvested at necropsy. **(B)** Corresponding adrenal weights. **(C)** Interscapular brown adipose tissue (BAT) collected at necropsy was expressed as a percent of body weight. *indicates significantly different from VEH/CON (*p<0.05, **p<0.01, ****p<0.001); ^indicates significantly different from L-DE-71 (^*p* < 0.05). Bars and error bars represent values expressed as mean ± s.e.m. n=5-8/group for epinephrine and *n*=8-21/group for BAT. [*Permission obtained for reuse of panel in* **Figure 1B** *, Kozlova et al, 2022, Front. Endocrinol. 13: 997304]*.

Due to the important role of epinephrine in glucose and lipid homeostasis, we examined whether adrenal content of epinephrine was altered by developmental exposure to DE-71. Adrenal epinephrine levels were similar to those reported previously for male wildtype C57Bl/6 mice using a radioenzymatic assay and LC-MS measurement instead of fluoroscopy (46). **Figure 6A** shows that L- and H-DE-71 exposure significantly elevated adrenal epinephrine (One-way ANOVA: *Exposure effect* F_(2,18)_=16.06, *p<.0001*; Tukey’s *post-hoc* VEH/CON vs L-DE-71 *p*=0.0001, VEH/CON vs H-DE-71 *p*=0.002). Adrenal weights were not different across experimental groups (**Figure 6B**). Because diabetes and fasting glucose level is associated with brown adipose tissue (BAT) activity (47), we measured BAT mass (47). When normalized to body weight, mean interscapular BAT mass was dramatically decreased by 30.2% in L-DE-71 relative to VEH/CON (One-way ANOVA: *Exposure effect* F_(2,41)_=6.82, *p*=0.061; Tukey’s *post-hoc* VEH/CON vs L-DE-71 *p*=0.01, L-DE-71 vs H-DE-71 *p*=0.01) (**Figure 6C**).

### DE-71 exposure alters hepatic glutamate dehydrogenase

**Figure 7:**
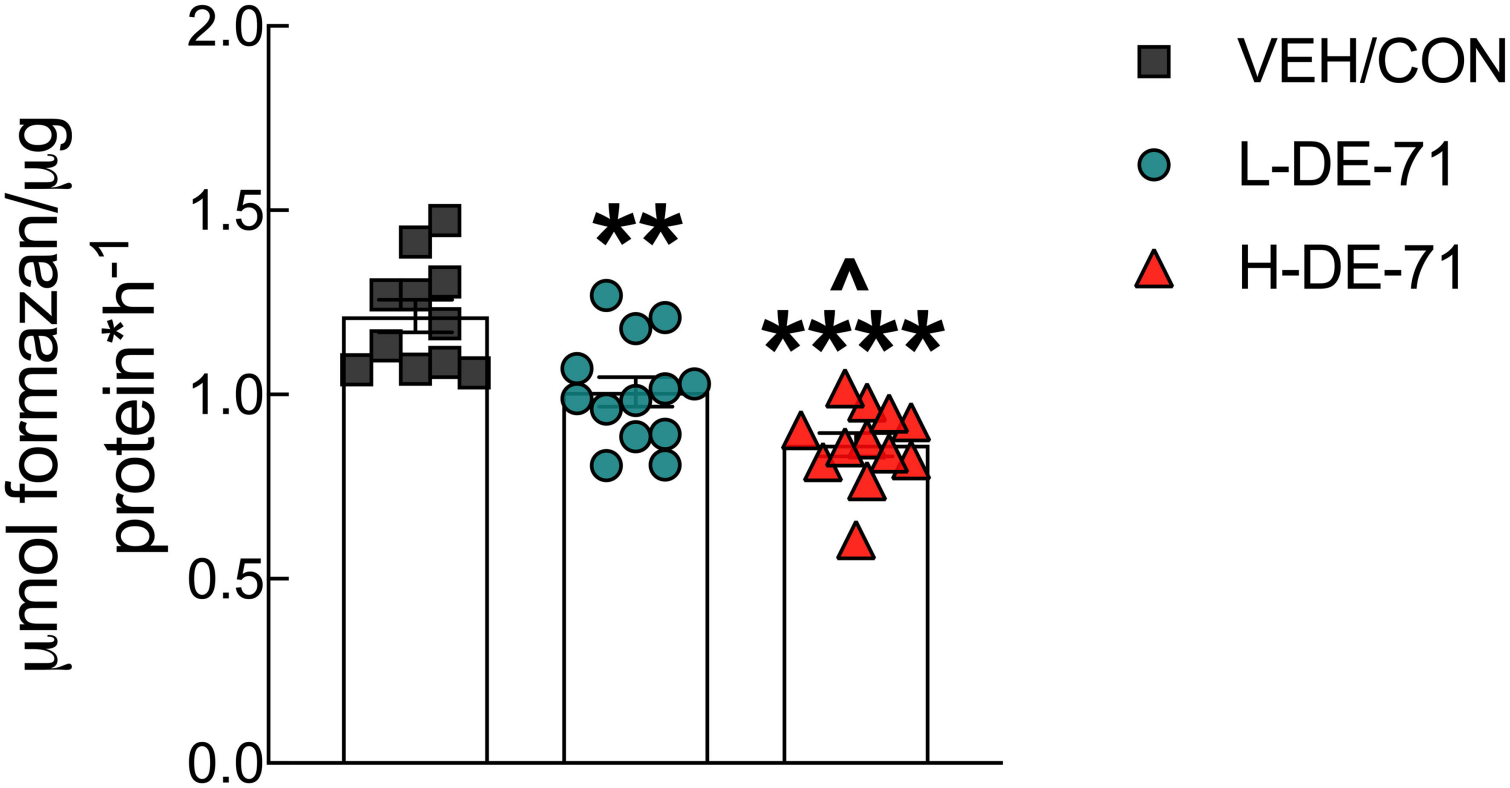
Effects of DE-71 on hepatic glutamate dehydrogenase (GDH). DE-71 exposure decreased GDH activity in a dose-dependent manner. *indicates significantly different from VEH/CON (**p < 0.01, ****p<0.0001); ^indicates significantly different from L-DE-71 (^*p* < 0.05). Bars and error bars represent values expressed as mean ± s.e.m. *n*=11-13/group.

Exposure to DE-71 decreases glucose tolerance which may be due to enhanced hepatic glucose production. To examine this possibility, we tested the hypothesis that PBDEs increase the activity of GDH a hepatic gluconeogenic enzyme. We found that exposure to L- and H-DE-71 significantly *reduced* enzymatic activity of GDH (One-way ANOVA: *Exposure effect* F_(2,33)_=19.51, *p<.0001*; Dunnett’s *post-hoc* VEH/CON vs L-DE-71 *p*=0.002, VEH/CON vs H-DE-71 *p*<0.0001) (**Figure 7**). The effect of DE-71 was dose-dependent on this metric (L-DE-71 vs H-DE-71 *p*=0.03).

### DE-71 exposure does not alter hepatic levels of endocannabinoids

**Figure 8:**
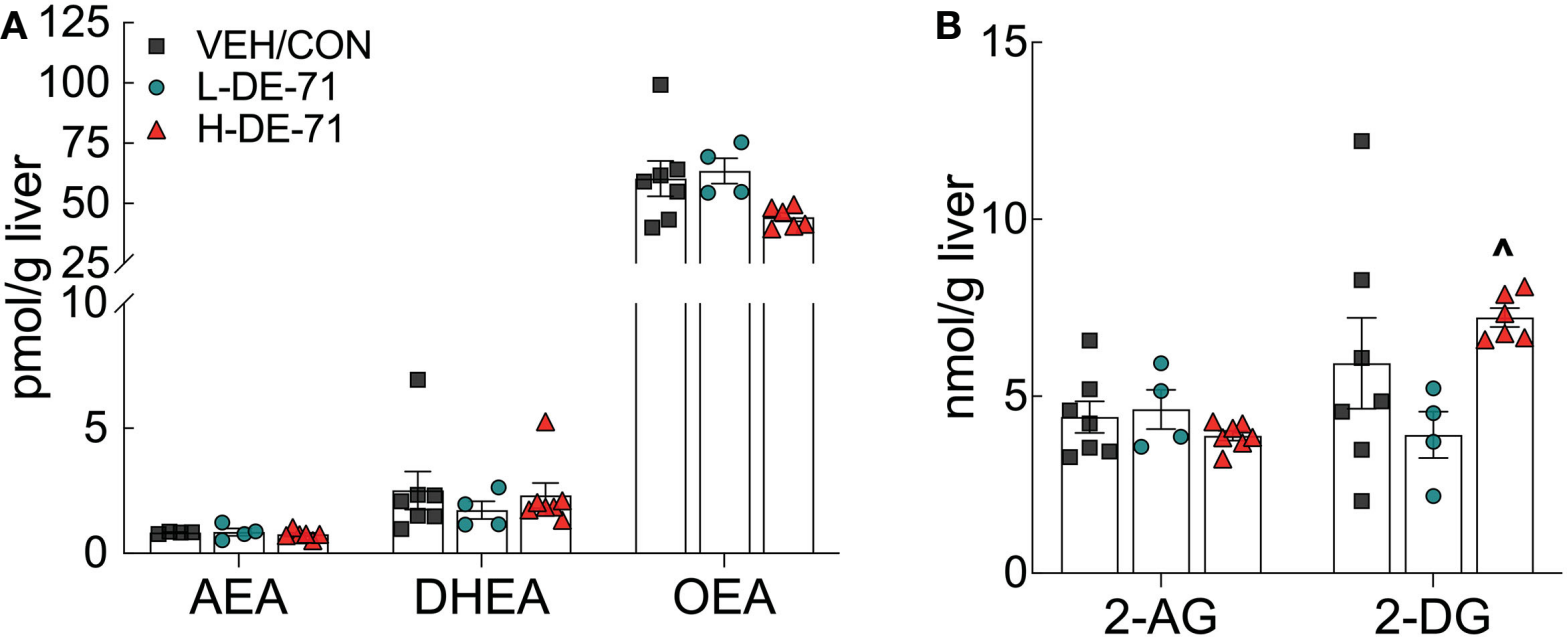
Hepatic levels of endocannabinoid (EC) and related fatty acid ethanolamides in DE-71 exposed male mice. *Post mortem* liver tissue was analyzed using UPLC/MS/MS. **(A)** AEA, DHEA and OEA. **(B)** 2-AG and 2-DG. ^indicates significantly different from L-DE-71 (^p < 0.05). Bars and error bars represent values expressed as mean ± s.e.m. *n*, 4-7/group. AEA, arachidonoylethanolamide (Anandamide); DHEA, docosahexanoyl ethanolamide; OEA, n-oleoyl ethanolamide; 2-AG, 2-arachidonoyl-*sn*-glycerol; 2-DG, monoacylglycerol 2-docosahexaenoyl-*sn*-glycerol.

Male F1 mice exposed to either dose of DE-71 displayed normal liver levels of the endocannabinoid (EC), anandamide (arachidonoylethanolamide, AEA), and related fatty acid ethanolamides, docosahexaenoyl ethanolamide (DHEA) and oleoylethanolamide (OEA), when compared to VEH/CON (**Figure 8A**). Fewer samples were used for LC-MS analysis of ECs than for other tests. This was due to high precision afforded by this method and to control for cost. No changes were detected for the other primary EC, 2-arachidonoyl-sn-glycerol (2-AG), and related monoacylglycerol, 2-docosahexaenoyl-sn-glycerol (2-DG) (**Figure 8B**).

## Discussion

Mounting evidence implicates environmental toxicants in increased susceptibility to obesity and diabetes (48, 49). Organohalogen compounds used indoors, such as brominated flame retardants, are suspected to contribute to these and other diseases (50, 51). Previous findings from our lab demonstrate that female siblings of the male offspring reported here, display a diabetogenic phenotype when exposed developmentally to an industrial penta-mixture of PBDEs, DE-71 (31). In this article, we continue our examination of the relationship between early-life PBDEs and regulation of glucose homeostasis by examining DE-71-exposed male offspring. Here, we show that developmental DE-71 exposure produces altered glucose tolerance, glucose response after insulin challenge, levels of glucoregulatory pancreatic and gut hormones and markers of liver glucose production and sympathetic activity. Although PBDEs in DE-71 act as MDCs in developmentally exposed male offspring, the altered metabolic profile observed in DE-71 exposed males does not exactly overlap with what was reported for exposed female offspring (31). Metrics examined in this study were chosen based on *in vivo* and *ex vivo* biomarkers of diabetes in adulthood that are used to validate diabetic animals models (52).

### Human relevance of the PBDE dosing paradigm

In our past works, we have demonstrated that exposure of mouse dams to 0.1 mg/kg/d and 0.4 mg/kg/d resulted in liver accumulation of ~3.2 ppm PBDEs in dam liver. This also resulted in PBDE levels in lipid weight of 0.2-1.0 ppm in the liver and 0.07-0.3 ppm in brains of exposed female offspring (31, 32). The liver is a key organ for glucose homeostasis and lipid metabolism as well as xenobiotic metabolism, while PBDE penetration into the brain may cause central disruption of glucoregulatory circuits (29). Liver PBDE levels in mice offspring are in range with maximum plasma levels reported in North American populations of Canadian indigenous Inuits and Crees (219–402 ng/g lipid wt) (21) and Californian women (~749.7 ng/g lipid wt) (53). In human studies, sum PBDE levels in children have been reported in the range of 43-366 ng/g lipid weight (54–56). Studies in toddlers report plasma ∑PBDE values with a range of 0.1-0.5 ppm (54, 55). Therefore, our dosing regimen produces a PBDE body burden that is similar to the levels experienced by certain human populations. In comparison, the EPA reference dose, the minimum dose that does not produce bioactivity, for DE-71 is 2x10^-3^ mg/kg/day or 2 ppb/day (57). Notably, using perinatal exposure to DE-71 the greatest body burden is observed in postnatal offspring (PND 15) and a 150-200-fold drop-off in PBDE levels is seen in adult offspring (PND 110) (32). This is of significance since it occurs during early postnatal development when biological programming has been shown to be more susceptible to endocrine disruption and neurotoxicity (58).

A few human studies have examined associations between PBDEs and metabolic syndrome/diabetes. Examining Chinese men and women, Zhang and others, found an apparent but not significant association between BDE-47 and T2D (25). Another study examined over 1000 cases showing significant risk produced by BDE-153 but did not report results by sex (22). Helaleh and others studied both males and females but sample sizes were very small (pilot) and data sets were combined, precluding analysis on the basis of sex (19). There are much fewer animal reports on the glucose metabolic effects of PBDEs and they lack sex-dependent reporting (25–27). Therefore, more research is needed to understand the association between metabolic disorders and environmentally relevant BDE mixtures, especially when exposure is developmental.

### Fasting blood glucose and glucose intolerance

In total, changes in glycemia metrics displayed by exposed male offspring represent the complex actions of PBDEs on the broad network of systems participating in the regulation of glucose metabolism (59, 60). Extended fasting yielded exaggerated hypoglycemia in DE-71 male offspring relative to VEH/CON. This was not seen after a 9 h fast needed for the subsequent ITT performed 1 week later, possibly due to the shorter fast duration or less likely due to potential metabolic alterations due to the prior fast. Results of the GTT conducted after an 11 h fast showed that H-DE-71 exposure produces dramatic glucose intolerance as compared to VEH/CON. Interestingly, this phenotype was most prominent at H-DE-71 although L-DE-71 male offspring showed incomplete glucose clearance and/or utilization. These results suggest differential metabolic disrupted phenotypes produced by DE-71 at 0.1 and 0.4 mg/kg. Despite the slight difference in dose of L- and H-DE-71, the chronic manner of exposure paradigm as well as the developmental timing may combine to augment responses in H-DE-71 to exogenous glucose challenge. Different doses of DE-71 within this tight range have been previously shown to produce differential bioactivity (61). Indeed, in female offspring, we have previously shown that 0.1 mg/kg but not 0.4 mg/kg DE-71 produces glucose intolerance in GTT (31). In fact, a non-linear dose response has also been commonly reported on behavioral and cellular/molecular processes affected by PBDEs (32, 62).

### Altered responses to insulin challenge

An ITT was used to examine insulin responsiveness in DE-71 exposed male offspring. In these experiments, developmental exposure to DE-71 at 0.1 mg/kg produced a significant reduction in recovery of plasma glucose clearance after 90-120 min post insulin i.p. challenge compared to VEH/CON. In contrast, no significant differences were observed in insulin K_ITT,_ measured within the first 30 min, suggesting normal glucose uptake after insulin challenge (insulin sensitivity). The combination of DE-71 effects - exaggerated fasting hypoglycemia, glucose intolerance and incomplete glucose clearance/utilization in response to insulin challenge demonstrates the complex actions of PBDEs on glucose homeostasis in males. Previously, we have observed abnormal glucose metabolism and other parameters in their DE-71-exposed female siblings (31). Together, with DE-71 induced endocrine-disrupting effects on glucoregulatory hormones (see below), maternal transfer of PBDEs in DE-71 during gestation and lactation produce metabolic disruption of their offspring that may lead to metabolic disorders in adulthood. In contrast, their common mothers displayed more subtle glucoregulatory alterations, suggesting that developing organisms are more susceptible to DE-71 (31). Taken together, our findings demonstrating prominent metabolic disturbances in adulthood after early-life, but not adult exposure support the developmental basis for the adult disease hypothesis described for metabolic and neurodevelopmental disorders (63).

### Deregulated pancreatic and gut glucoregulatory hormones

In this study we showed that L-DE-71 males also display elevated glucoregulatory hormones GLP-1 and glucagon when compared to levels in VEH/CON. In contrast, insulin levels, which are sometimes altered in T2D (44), were similar across exposure groups. Glucagon secretion plays an essential role in the regulation of hepatic glucose production and may contribute to hyperglycemia typical of T2D. Gastrointestinal hormones like GLP-1, that are secreted in response to oral glucose, have incretin effects which can normalize glucose homeostasis. Studies employing the GLP-1 receptor antagonist, exendin 9-39 amide, indicate that endogenous GLP-1 performs important regulation over glucagon secretion during fasting and after a meal (64, 65). Clinical studies on T2D suggest that deregulation of GLP-1’s inhibitory effect on glucagon may be as important as GLP-1’s stimulatory effect on insulin secretion (45). However, in the L-DE-71 exposed males, GLP-1 was not reduced concomitant with elevated glucagon levels. The deregulation of GLP-1 and glucagon by PBDEs, may lead to opposing effects on glucose homeostasis, preventing the metabolic disruption seen in H-DE-71 mice which showed no significant changes in these glucoregulatory endocrines. Other effects of DE-71 at 0.4 mg/kg may be responsible for glucose intolerance seen only at this dose. Further study is necessary to determine the metabolic consequences of PBDE effects on individual hormones.

### Comparison to female glucoregulatory phenotype

**Figure 9:**
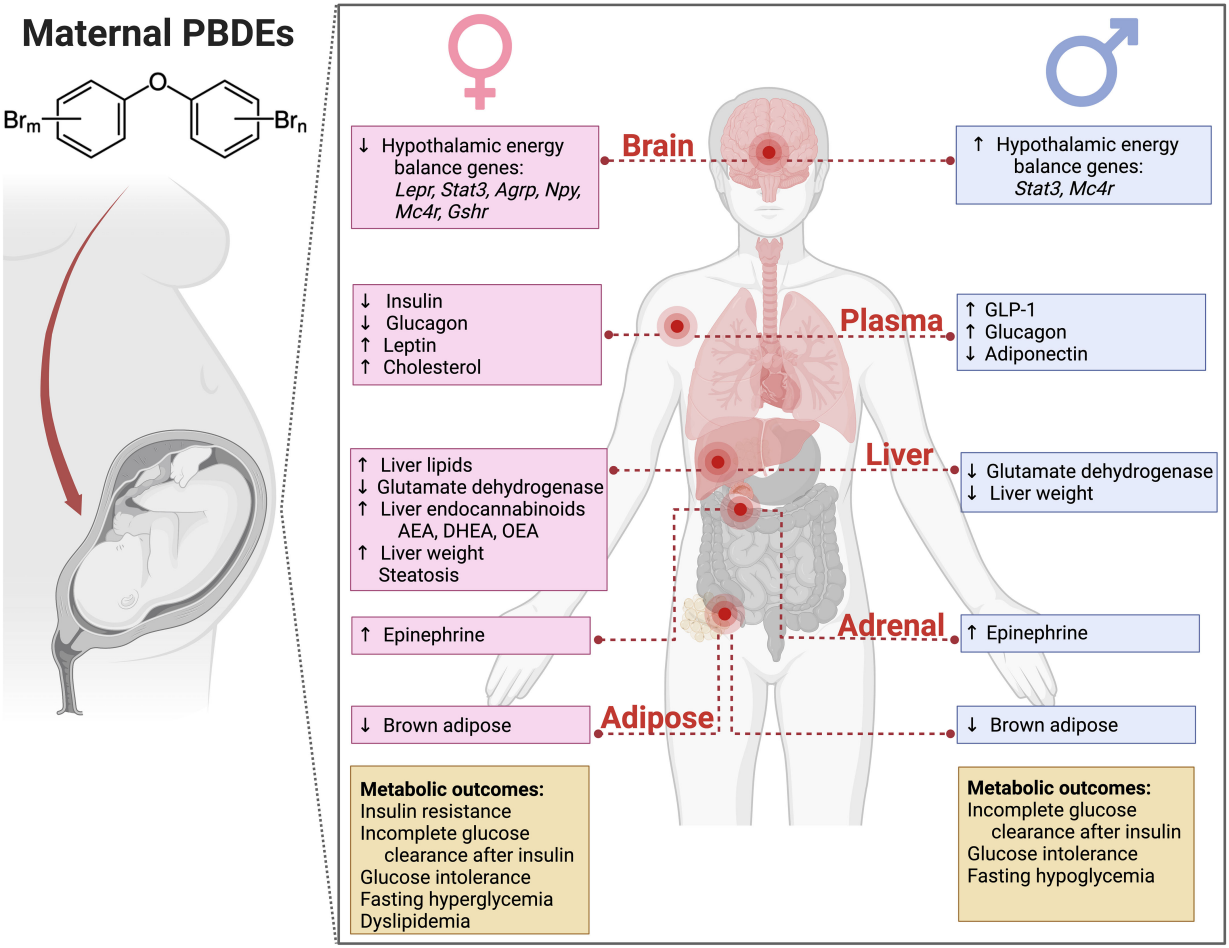
Summary of physiological consequences in adulthood of developmental exposure to PBDEs in DE-71 (0.1 and 0.4 mg/kg/d, GD0-PND21). Maternal DE-71 exposure produces sex-dependent differences in adult mice offspring. Offspring were exposed to DE-71 *via* the dam during the *in utero* and lactational period. Adult offspring were evaluated using *in vivo* glucose and insulin responses and ex vivo tissue analyses. The glucoregulation results obtained from exposure female offspring and lipid metrics measured in exposed female and male offspring have been previously reported (28, 29).

The glucose metabolic profile in male offspring that was altered by L- and H-DE-71 is not identical to that seen in L-DE-71 exposed female offspring. Specifically, we detected sexually dimorphic differences in glucose tolerance and glycemic response to insulin challenge. L-DE-71 exposed females showed fasting hyperglycemia, glucose intolerance, insulin resistance, decreased plasma insulin, and elevated hepatic endocannabinoids (31). In contrast, perinatal DE-71 exposure in males resulted in fasting hypoglycemia, incomplete glucose clearance/utilization in response to insulin, and elevated glucagon and GLP-1 (**Figure 9**). Moreover, H-DE-71 males displayed glucose intolerance. In contrast, Wang and others did not detect an abnormal response to glucose in male mice exposed only to one congener BDE-47 at 0.002-0.2 mg/kg BDE-47 (26). The differential abnormal glucoregulatory profiles observed in male mice exposed to DE-71 reinforce our previous findings showing sexual dimorphism in the regulation of lipid metabolism in exposed male and female offspring (29). Some actions of L-DE-71 (but not H-DE-71) were also observed in our report on exposed female, i.e., reduced hepatic glutamate dehydrogenase enzymatic activity, elevated adrenal epinephrine, and reduced thermogenic brown adipose tissue mass (31) contributions of these systems to glucose homeostasis (31, 66).

### DE-71 alters hepatic brown adipose and adrenal parameters regulating glucose homeostasis

Glutamate dehydrogenase activity measured in exposed male livers was reduced in L- and H-DE-71 and suggests altered glucose metabolism. Other studies have shown PBDE-induced alterations in hepatic glucolipid regulatory and exposome gene expression. For example, rats exposed to DE-71, made up primarily of BDE-47 and BDE-99, as well as other minor constituents, exhibited changes in liver gene expression patterns that matched those associated with metabolic syndrome (67, 68). Our reports describing the metabolic dyshomeostasis in adult male and female mice exposed developmentally to DE-71 warn against the addition of harmful flame retardants to home and personal products. PBDEs can penetrate human serum and breastmilk and transfer efficiently to developing offspring (7–9). The long-lasting changes in glucose and lipid metabolism may be a risk factor for developing T2D and liver steatosis, a clinical condition comorbid with MetS with increasing prevalence in the adult US population (69).

Brown adipose tissue (BAT) activity increases energy expenditure by burning fat and increasing metabolic rate and can utilize glucose especially when stimulated by insulin (70). Therefore, BAT adipocytes contribute to glucose homeostasis and their activity has been inversely associated with diabetes (47). Here we report reduced BAT mass in L-DE-71 males offspring relative to VEH/CON, which may have implications for altered glucose homeostasis. Lower BAT mass impairs glucose clearance (47) and is negatively associated with central obesity and diabetes (71–73). Since BAT is under the trophic influence of ß-adrenergic sympathetic- and insulin-mediated regulation and epinephrine (71–73), it is important to note that PBDE exposure also produced elevated adrenal epinephrine. Our findings suggest that altered BAT mass may be due to enhanced sympathetic activity and/or insulin responses but further study is needed to clarify this relationship. The sympathoadrenal nervous system exerts regulatory control over nutrient release and utilization. Adrenal epinephrine, which is regulated by and responds to sympathetic nervous system activation, was elevated in L- and H-DE-71, a profile that is characteristic of MetS (74). Interestingly, DE-71 exposure produced similar effects in exposed female offspring (29). This study should motivate the future research of toxicological mechanisms of BDEs.

### Developmental PBDE exposure produces a more robust abnormal metabolic phenotype

It is important to note that developmental exposure to DE-71 ends at weaning, but that dysregulated glucoregulatory phenotypes in exposed male and female offspring are observed in adulthood (31). We speculate that PBDEs are acting as MDCs by altering the function of several organ systems during early development and reprogramming physiological control systems during early life. Previously, we have found that tissue BDE burden (liver and brain) is greatest during postnatal development (P30) and decreases to lower levels over the first 4 months into adult life [see (28, 32)]. However, PBDE body burden is still significantly greater in adult exposed vs adult control mice, and it is possible that PBDE levels in adult tissues continue to cause effects throughout life. Nevertheless, we have demonstrated that the glucolipid status that develops after adult exposure is much milder compared to that after gestational/lactational exposure (28). This suggests that there is a critical window in early life (fetal and/or postnatal) that makes developing organisms more susceptible to metabolic disrupting effects of PBDEs. This life stage has been reported by others to be especially vulnerable to neurobehavioral and metabolic toxicity by organohalogens (28, 75–78). For these reasons, we favor the developmental origins of health and disease (DOHaD) hypothesis in explaining the metabolic disruptive effects of perinatal exposure to DE-71, which emphasizes the reduced detoxification capacity of developing organisms and the critical processes underlying growth and differentiation during development (79–81). Of course it is likely that PBDEs continue to cause havoc on homeostatic and other physiological systems as they generate responses to their natural stimuli (35, 82–84).

## Conclusions

Altogether, the current results determined from *in vivo* and *ex vivo* parameters measured in DE-71-exposed male offspring indicate broad, complex effects of developmental exposure to PBDEs on glucose homeostasis and glucoregulatory parameters involving various organ systems. Our results support human studies reporting the positive association between body burdens of PBDEs and T2D and MetS (22). Importantly, our results warn that maternal transfer of PBDEs can produce metabolic and endocrine disruption of developing male offspring that persists into adulthood, at which time they may contribute to metabolic diseases. Both exposed male and female offspring are susceptible, albeit they display different and complex phenotypes. Our findings should be leveraged develop inform risk management practices, provide best treatments in the clinic and improve consistency in experimental design studies.

## Data availability statement

The raw data supporting the conclusions of this article will be made available by the authors, without undue reservation.

## Ethics statement

The animal study was reviewed and approved by Care and treatment of animals was performed in accordance with guidelines from and approved by the University of California, Riverside Institutional Animal Care and Use Committee (IACUC) (AUP# 20170026 and 20200018).

## Author contributions

Conceptualization, MC-C, EK. Methodology, MC-C, EK, ND, JK, BC. Validation, MC-C, EK, BC, PP, ND. Formal Analysis, EK, MC-C, PP, ND. Investigation, EK, BC, PP, JK, AB, MC-C, MD. Writing – Original Draft, EK, MC-C, AB. Writing – Review & Editing, MC-C, EK, AB. Visualization, EK. Resources, MC-C., ND. Data Curation, EK, MC-C. Supervision, MC-C, EK, ND. Project Administration, MC-C, EK. Funding Acquisition, MC-C, EK, ND. All authors contributed to the article and approved the submitted version.

## Author disclaimer

The opinions and assertions expressed herein are those of the author(s) and do not necessarily reflect the official policy or position of the Uniformed Services University or the Department of Defense. J.K. is now a 2nd Lieutenant at the Uniformed Services University, Department of Defense. Her work was performed at the University of California, Riverside before becoming a military officer. However, we want to emphasize that the opinions and assertions expressed herein are those of the authors and do not necessarily reflect the official policy or position of the Uniformed Services University or the Department of Defense.

## References

[1] Papalou O Kandaraki EA Papadakis G Diamanti-Kandarakis E. Endocrine disrupting chemicals: An occult mediator of metabolic disease. Front Endocrinol (2019) 10:112. doi: 10.3389/fendo.2019.00112 PMC6406073 30881345

[2] Siddiqi MA Laessig RH Reed KD. Polybrominated diphenyl ethers (PBDEs): New pollutants-old diseases. Clin Med Res (2003) 1:281–90. doi: 10.3121/cmr.1.4.281 PMC1069057 15931321

[3] Harley KG Marks AR Chevrier J Bradman A Sjödin A Eskenazi B. PBDE concentrations in women’s serum and fecundability. Environ Health Perspect (2010) 118:699–704. doi: 10.1289/ehp.0901450 20103495 PMC2866688

[4] Zhang T Zhou X Xu A Tian Y Wang Y Zhang Y. Toxicity of polybrominated diphenyl ethers (PBDEs) on rodent male reproductive system: A systematic review and meta-analysis of randomized control studies. Sci Total Environ (2020) 720:137419. doi: 10.1016/j.scitotenv.2020.137419 32325560

[5] Herbstman JB Sjödin A Kurzon M Lederman SA Jones RS Rauh V. Prenatal exposure to PBDEs and neurodevelopment. Environ Health Perspect (2010) 118:712–9. doi: 10.1289/ehp.0901340 PMC2866690 20056561

[6] Gilbert ME Rovet J Chen Z Koibuchi N. Developmental thyroid hormone disruption: Prevalence, environmental contaminants and neurodevelopmental consequences. Neurotoxicology (2012) 33:842–52. doi: 10.1016/j.neuro.2011.11.005 22138353

[7] Vizcaino E Grimalt JO Fernández-Somoano A Tardon A. Transport of persistent organic pollutants across the human placenta. Environ Int (2014) 65:107–15. doi: 10.1016/j.envint.2014.01.004 24486968

[8] Choi G Kim S Kim S Kim S Choi Y Kim H-J. Occurrences of major polybrominated diphenyl ethers (PBDEs) in maternal and fetal cord blood sera in Korea. Sci Total Environ (2014) 491-492:219–26. doi: 10.1016/j.scitotenv.2014.02.071 24636800

[9] Gómara B Herrero L Ramos JJ Mateo JR Fernández MA García JF. Distribution of polybrominated diphenyl ethers in human umbilical cord serum, paternal serum, maternal serum, placentas, and breast milk from Madrid population, Spain. Environ Sci Technol (2007) 41:6961–8. doi: 10.1021/es0714484 17993135

[10] Abbasi G Li L Breivik K. Global historical stocks and emissions of PBDEs. Environ Sci Technol (2019) 53:6330–40. doi: 10.1021/acs.est.8b07032 31083912

[11] Zhang Z Sun Z-Z Xiao X Zhou S Wang X-C Gu J. Mechanism of BDE209-induced impaired glucose homeostasis based on gene microarray analysis of adult rat liver. Arch Toxicol (2013) 87:1557–67. doi: 10.1007/s00204-013-1059-8 23640034

[12] Feng C Xu Y Zha J Li J Wu F Wang Z. Metabolic pathways of decabromodiphenyl ether (BDE209) in rainbow trout (Oncorhynchus mykiss) *via* intraperitoneal injection. Environ Toxicol Pharmacol (2015) 39:536–44. doi: 10.1016/j.etap.2015.01.006 25681704

[13] Huwe JK Smith DJ. Accumulation, whole-body depletion, and debromination of decabromodiphenyl ether in male sprague-dawley rats following dietary exposure. Environ Sci Technol (2007) 41:2371–7. doi: 10.1021/es061954d 17438789

[14] Wang F Wang J Dai J Hu G Wang J Luo X. Comparative tissue distribution, biotransformation and associated biological effects by decabromodiphenyl ethane and decabrominated diphenyl ether in male rats after a 90-day oral exposure study. Environ Sci Technol (2010) 44:5655–60. doi: 10.1021/es101158e 20536227

[15] Ogurtsova K da Rocha Fernandes JD Huang Y Linnenkamp U Guariguata L Cho NH. IDF Diabetes Atlas: Global estimates for the prevalence of diabetes for 2015 and 2040. Diabetes Res Clin Pract (2017) 128:40–50. doi: 10.1016/j.diabres.2017.03.024 28437734

[16] Bommer C Sagalova V Heesemann E Manne-Goehler J Atun R Bärnighausen T. Global economic burden of diabetes in adults: Projections from 2015 to 2030. Diabetes Care (2018) 41:963–70. doi: 10.2337/dc17-1962 29475843

[17] Yan D Jiao Y Yan H Liu T Yan H Yuan J. Endocrine-disrupting chemicals and the risk of gestational diabetes mellitus: A systematic review and meta-analysis. Environ Health (2022) 21:53. doi: 10.1186/s12940-022-00858-8 35578291 PMC9109392

[18] Han X Meng L Li Y Li A Turyk ME Yang R. Associations between the exposure to persistent organic pollutants and type 2 diabetes in East China: A case-control study. Chemosphere (2020) 241:125030. doi: 10.1016/j.chemosphere.2019.125030 31606000

[19] Helaleh M Diboun I Al-Tamimi N Al-Sulaiti H Al-Emadi M Madani A. Association of polybrominated diphenyl ethers in two fat compartments with increased risk of insulin resistance in obese individuals. Chemosphere (2018) 209:268–76. doi: 10.1016/j.chemosphere.2018.06.108 29933163

[20] Ongono JS Dow C Gambaretti J Severi G Boutron-Ruault M-C Bonnet F. Dietary exposure to brominated flame retardants and risk of type 2 diabetes in the French E3N cohort. Environ Int (2019) 123:54–60. doi: 10.1016/j.envint.2018.11.040 30496982

[21] Cordier S Anassour-Laouan-Sidi E Lemire M Costet N Lucas M Ayotte P. Association between exposure to persistent organic pollutants and mercury, and glucose metabolism in two Canadian indigenous populations. Environ Res (2020) 184:109345. doi: 10.1016/j.envres.2020.109345 32172074

[22] Lim J-S Lee D-H Jacobs DR. Association of brominated flame retardants with diabetes and metabolic syndrome in the U.S. population, 2003–2004. Diabetes Care (2008) 31:1802–7. doi: 10.2337/dc08-0850 PMC2518348 18559655

[23] Baumert BO Goodrich JA Hu X Walker DI Alderete TL Chen Z. Plasma concentrations of lipophilic persistent organic pollutants and glucose homeostasis in youth populations. Environ Res (2022) 212:113296. doi: 10.1016/j.envres.2022.113296 35447156 PMC9831292

[24] Suvorov A Naumov V Shtratnikova V Logacheva M Shershebnev A Wu H. Rat liver epigenome programing by perinatal exposure to 2,2’,4'4'-tetrabromodiphenyl ether. Epigenomics (2020) 12:235–49. doi: 10.2217/epi-2019-0315 31833787

[25] Zhang Z Li S Liu L Wang L Xiao X Sun Z. Environmental exposure to BDE47 is associated with increased diabetes prevalence: Evidence from community-based case-control studies and an animal experiment. Sci Rep (2016) 6:27854. doi: 10.1038/srep27854 27291303 PMC4904204

[26] Wang D Yan J Teng M Yan S Zhou Z Zhu W. *In utero* and lactational exposure to BDE-47 promotes obesity development in mouse offspring fed a high-fat diet: Impaired lipid metabolism and intestinal dysbiosis. Arch Toxicol (2018) 92:1847–60. doi: 10.1007/s00204-018-2177-0 29523931

[27] McIntyre RL Kenerson HL Subramanian S Wang SA Kazami M Stapleton HM. Polybrominated diphenyl ether congener, BDE-47, impairs insulin sensitivity in mice with liver-specific pten deficiency. BMC Obes (2015) 2:3. doi: 10.1186/s40608-014-0031-3 26217518 PMC4510911

[28] Kozlova EV Chinthirla BD Pérez PA DiPatrizio NV Argueta DA Phillips AL. Maternal transfer of environmentally relevant polybrominated diphenyl ethers (PBDEs) produces a diabetic phenotype and disrupts glucoregulatory hormones and hepatic endocannabinoids in adult mouse female offspring. Sci Rep (2020) 10:18102. doi: 10.1038/s41598-020-74853-9 33093533 PMC7582149

[29] Kozlova EV Denys ME Benedum J Valdez MC Enriquez D Bishay AE. Developmental exposure to indoor flame retardants and hypothalamic molecular signatures: Sex-dependent reprogramming of lipid homeostasis. Front Endocrinol (2022) 13:997304. doi: 10.3389/fendo.2022.997304 PMC9580103 36277707

[30] Wu Z He C Han W Song J Li H Zhang Y. Exposure pathways, levels and toxicity of polybrominated diphenyl ethers in humans: A review. Environ Res (2020) 187:109531. doi: 10.1016/j.envres.2020.109531 32454306

[31] Kozlova EV Chinthirla BD Pérez PA DiPatrizio NV Argueta DA Phillips AL. Maternal transfer of environmentally relevant polybrominated diphenyl ethers (PBDEs) produces a diabetic phenotype and disrupts glucoregulatory hormones and hepatic endocannabinoids in adult mouse female offspring. Sci Rep (2020) 10:18102. doi: 10.1038/s41598-020-74853-9 33093533 PMC7582149

[32] Kozlova EV Valdez MC Denys ME Bishay AE Krum JM Rabbani KM. Persistent autism-relevant behavioral phenotype and social neuropeptide alterations in female mice offspring induced by maternal transfer of PBDE congeners in the commercial mixture DE-71. Arch Toxicol (2022):335–65. doi: 10.1007/s00204-021-03163-4 PMC8536480 34687351

[33] De León DD Crutchlow MF Ham J-YN Stoffers DA. Role of glucagon-like peptide-1 in the pathogenesis and treatment of diabetes mellitus. Int J Biochem Cell Biol (2006) 38:845–59. doi: 10.1016/j.biocel.2005.07.011 16202636

[34] Godoy-Matos AF. The role of glucagon on type 2 diabetes at a glance. Diabetol Metab Syndr (2014) 6:91. doi: 10.1186/1758-5996-6-91 25177371 PMC4148933

[35] Hoppe AA Carey GB. Polybrominated diphenyl ethers as endocrine disruptors of adipocyte metabolism. Obesity (2007) 15:2942–50. doi: 10.1038/oby.2007.351 18198302

[36] Legato MJ Gelzer A Goland R Ebner SA Rajan S Villagra V. Writing group for the partnership for gender-specific medicine. gender-specific care of the patient with diabetes: Review and recommendations. Gend Med (2006) 3:131–58. doi: 10.1016/s1550-8579(06)80202-0 16860272

[37] Arnetz L Ekberg NR Alvarsson M. Sex differences in type 2 diabetes: Focus on disease course and outcomes. Diabetes Metab Syndr Obes (2014) 7:409–20. doi: 10.2147/DMSO.S51301 PMC4172102 25258546

[38] Drage DS Heffernan AL Cunningham TK Aylward LL Mueller JF Sathyapalan T. Serum measures of hexabromocyclododecane (HBCDD) and polybrominated diphenyl ethers (PBDEs) in reproductive-aged women in the united kingdom. Environ Res (2019) 177:108631. doi: 10.1016/j.envres.2019.108631 31404810

[39] Perez PA DiPatrizio NV. Impact of maternal western diet-induced obesity on offspring mortality and peripheral endocannabinoid system in mice. PLos One (2018) 13:e0205021. doi: 10.1371/journal.pone.0205021 30273406 PMC6166980

[40] Wiley MB DiPatrizio NV. Diet-induced gut barrier dysfunction is exacerbated in mice lacking cannabinoid 1 receptors in the intestinal epithelium. Int J Mol Sci (2022) 23:10549. doi: 10.3390/ijms231810549 36142461 PMC9504303

[41] Lee YP Lardy HA. Influence of Thyroid Hormones on l-α-Glycerophosphate Dehydrogenases and Other Dehydrogenases in Various Organs of the Rat. J Biol Chem (1965) 240:1427–36. doi: 10.1016/s0021-9258(18)97593-9 14284758

[42] Egido LLD Del Egido LL Navarro-Miró D Martinez-Heredia V Toorop PE. Iannetta PPM. a spectrophotometric assay for robust viability testing of seed batches using 2,3,5-triphenyl tetrazolium chloride: Using hordeum vulgare l. as a model. Front Plant Sci (2017) 8:747. doi: 10.3389/fpls.2017.00747 28559902 PMC5433298

[43] Kelner KL Levine RA Morita K Pollard HB. A comparison of trihydroxyindole and HPLC/electrochemical methods for catecholamine measurement in adrenal chromaffin cells. Neurochem Int (1985) 7:373–8. doi: 10.1016/0197-0186(85)90128-7 20492937

[44] Wysham C Shubrook J. Beta-cell failure in type 2 diabetes: Mechanisms, markers, and clinical implications. Postgrad Med (2020) 132:676–86. doi: 10.1080/00325481.2020.1771047 32543261

[45] Holst JJ Christensen M Lund A de Heer J Svendsen B Kielgast U. Regulation of glucagon secretion by incretins. Diabetes Obes Metab (2011) 13 Suppl 1:89–94. doi: 10.1111/j.1463-1326.2011.01452.x 21824261

[46] Bao X Lu CM Liu F Gu Y Dalton ND Zhu B-Q. Epinephrine is required for normal cardiovascular responses to stress in the phenylethanolamine n-methyltransferase knockout mouse. Circulation (2007) 116:1024–31. doi: 10.1161/CIRCULATIONAHA.107.696005 17698731

[47] Lee P Greenfield JR Ho KKY Fulham MJ. A critical appraisal of the prevalence and metabolic significance of brown adipose tissue in adult humans. Am J Physiol Endocrinol Metab (2010) 299:E601–6. doi: 10.1152/ajpendo.00298.2010 20606075

[48] Ribeiro CM Beserra BTS Silva NG Lima CL Rocha PRS Coelho MS. Exposure to endocrine-disrupting chemicals and anthropometric measures of obesity: A systematic review and meta-analysis. BMJ Open (2020) 10:e033509. doi: 10.1136/bmjopen-2019-033509 PMC7311014 32565448

[49] Lind PM Lind L. Endocrine-disrupting chemicals and risk of diabetes: An evidence-based review. Diabetologia (2018) 61:1495–502. doi: 10.1007/s00125-018-4621-3 PMC6445457 29744538

[50] Kodavanti PRS Stoker TE Fenton SE Curras-Collazo M. Chapter 36 - brominated flame retardants. In: Gupta RC, editor. Reproductive and developmental toxicology, 3rd ed. Academic Press (2022). p. 691–726. doi: 10.1016/B978-0-323-89773-0.00036-9

[51] Kodavanti PRS Curras-Collazo MC. Neuroendocrine actions of organohalogens: Thyroid hormones, arginine vasopressin, and neuroplasticity. Front Neuroendocrinol (2010) 31:479–96. doi: 10.1016/j.yfrne.2010.06.005 20609372

[52] King AJF. The use of animal models in diabetes research. Br J Pharmacol (2012) 166:877–94. doi: 10.1111/j.1476-5381.2012.01911.x PMC3417415 22352879

[53] Hurley S Goldberg D Nelson DO Guo W Wang Y Baek H-G. Temporal evaluation of polybrominated diphenyl ether (PBDE) serum levels in middle-aged and older California women, 2011–2015. Environ Sci Technol (2017) 51:4697–704. doi: 10.1021/acs.est.7b00565 PMC5526086 28304169

[54] Fischer D Hooper K Athanasiadou M Athanassiadis I Bergman A. Children show highest levels of polybrominated diphenyl ethers in a California family of four: A case study. Environ Health Perspect (2006) 114:1581–4. doi: 10.1289/ehp.8554 PMC1626410 17035146

[55] Stapleton HM Eagle S Sjödin A Webster TF. Serum PBDEs in a north Carolina toddler cohort: Associations with handwipes, house dust, and socioeconomic variables. Environ Health Perspect (2012) 120:1049–54. doi: 10.1289/ehp.1104802 PMC3404669 22763040

[56] Schecter A Päpke O Tung KC Joseph J Harris TR Dahlgren J. Polybrominated diphenyl ether flame retardants in the U.S. population: Current levels, temporal trends, and comparison with dioxins, dibenzofurans, and polychlorinated biphenyls. J Occup Environ Med (2005) 47:199. doi: 10.1097/01.jom.0000158704.27536.d2 15761315

[57] Pentabromodiphenyl ether CASRN 32534-81-9. Available at: https://iris.epa.gov/ChemicalLanding/&substance_nmbr=184 (Accessed December 4, 2022).

[58] Schug TT Blawas AM Gray K Heindel JJ Lawler CP. Elucidating the links between endocrine disruptors and neurodevelopment. Endocrinology (2015) 156:1941–51. doi: 10.1210/en.2014-1734 PMC5393340 25714811

[59] Kowalski GM Bruce CR. The regulation of glucose metabolism: Implications and considerations for the assessment of glucose homeostasis in rodents. Am J Physiol Endocrinol Metab (2014) 307:E859–71. doi: 10.1152/ajpendo.00165.2014 25205823

[60] Aronoff SL Berkowitz K Shreiner B Want L. Glucose metabolism and regulation: Beyond insulin and glucagon. Diabetes Spectr (2004) 17:183–90. doi: 10.2337/diaspect.17.3.183

[61] Suvorov A Girard S Lachapelle S Abdelouahab N Sebire G Takser L. Perinatal exposure to low-dose BDE-47, an emergent environmental contaminant, causes hyperactivity in rat offspring. Neonatology (2009) 95:203–9. doi: 10.1159/000155651 18799892

[62] Gillera SEA Marinello WP Horman BM Phillips AL Ruis MT Stapleton HM. Sex-specific effects of perinatal FireMaster® 550 (FM 550) exposure on socioemotional behavior in prairie voles. Neurotoxicol Teratol (2020) 79:106840. doi: 10.1016/j.ntt.2019.106840 31730801 PMC7214199

[63] Barker DJP. The developmental origins of adult disease. J Am Coll Nutr (2004) 23:588S–95S. doi: 10.1080/07315724.2004.10719428 15640511

[64] Edwards CM Todd JF Mahmoudi M Wang Z Wang RM Ghatei MA. Glucagon-like peptide 1 has a physiological role in the control of postprandial glucose in humans: Studies with the antagonist exendin 9-39. Diabetes (1999) 48:86–93. doi: 10.2337/diabetes.48.1.86 9892226

[65] Kielgast U Holst JJ Madsbad S. Antidiabetic actions of endogenous and exogenous GLP-1 in type 1 diabetic patients with and without residual β-cell function. Diabetes (2011) 60:1599–607. doi: 10.2337/db10-1790 PMC3292336 21441444

[66] Jensen TL Kiersgaard MK Sørensen DB Mikkelsen LF. Fasting of mice: A review. Lab Anim (2013) 47:225–40. doi: 10.1177/0023677213501659 24025567

[67] Sanders JM Burka LT Smith CS Black W James R Cunningham ML. Differential expression of CYP1A, 2B, and 3A genes in the F344 rat following exposure to a polybrominated diphenyl ether mixture or individual components. Toxicol Sci (2005) 88:127–33. doi: 10.1093/toxsci/kfi288 16107549

[68] Dunnick JK Brix A Cunny H Vallant M Shockley KR. Characterization of polybrominated diphenyl ether toxicity in wistar han rats and use of liver microarray data for predicting disease susceptibilities. Toxicol Pathol (2012) 40:93–106. doi: 10.1177/0192623311429973 22267650 PMC4816085

[69] Reed J Bain S Kanamarlapudi V. A review of current trends with type 2 diabetes epidemiology, aetiology, pathogenesis, treatments and future perspectives. Diabetes Metab Syndr Obes (2021) 14:3567–602. doi: 10.2147/DMSO.S319895 PMC8369920 34413662

[70] Maliszewska K Kretowski A. Brown adipose tissue and its role in insulin and glucose homeostasis. Int J Mol Sci (2021) 22:1530. doi: 10.3390/ijms22041530 33546400 PMC7913527

[71] Contreras C González-García I Martínez-Sánchez N Seoane-Collazo P Jacas J Morgan DA. Central ceramide-induced hypothalamic lipotoxicity and ER stress regulate energy balance. Cell Rep (2014) 9:366–77. doi: 10.1016/j.celrep.2014.08.057 PMC5157160 25284795

[72] Wang Q Zhang M Ning G Gu W Su T Xu M. Brown adipose tissue in humans is activated by elevated plasma catecholamines levels and is inversely related to central obesity. PLos One (2011) 6:e21006. doi: 10.1371/journal.pone.0021006 21701596 PMC3118816

[73] Sharara-Chami RI Joachim M Mulcahey M Ebert S Majzoub JA. Effect of epinephrine deficiency on cold tolerance and on brown adipose tissue. Mol Cell Endocrinol (2010) 328:34–9. doi: 10.1016/j.mce.2010.06.019 20619316

[74] Ziegler MG Elayan H Milic M Sun P Gharaibeh M. Epinephrine and the metabolic syndrome. Curr Hypertens Rep (2012) 14:1–7. doi: 10.1007/s11906-011-0243-6 22124970

[75] Dunnick JK Shockley KR Pandiri AR Kissling GE Gerrish KE Ton TV. PBDE-47 and PBDE mixture (DE-71) toxicities and liver transcriptomic changes at PND 22 after *in utero*/postnatal exposure in the rat. Arch Toxicol (2018) 92:3415–33. doi: 10.1007/s00204-018-2292-y PMC6706773 30206662

[76] Driscoll LL Gibson AM Hieb A. Chronic postnatal DE-71 exposure: effects on learning, attention and thyroxine levels. Neurotoxicol Teratol (2009) 31:76–84. doi: 10.1016/j.ntt.2008.11.003 19068229

[77] Costa LG de Laat R Tagliaferri S Pellacani C. A mechanistic view of polybrominated diphenyl ether (PBDE) developmental neurotoxicity. Toxicol Lett (2014) 230:282–94. doi: 10.1016/j.toxlet.2013.11.011 PMC4028440 24270005

[78] Coburn CG Watson-Siriboe A Hou B Cheetham C Gillard ER Lin L. Permanently compromised NADPH-diaphorase activity within the osmotically activated supraoptic nucleus after *in utero* but not adult exposure to aroclor 1254. Neurotoxicology (2015) 47:37–46. doi: 10.1016/j.neuro.2014.12.009 25572879

[79] Barker DJ. Fetal origins of coronary heart disease. BMJ (1995) 311:171–4. doi: 10.1136/bmj.311.6998.171 PMC2550226 7613432

[80] Treviño LS Katz TA. Endocrine disruptors and developmental origins of nonalcoholic fatty liver disease. Endocrinology (2018) 159:20–31. doi: 10.1210/en.2017-00887 29126168 PMC5761605

[81] Heindel JJ Vandenberg LN. Developmental origins of health and disease: A paradigm for understanding disease cause and prevention. Curr Opin Pediatr (2015) 27:248–53. doi: 10.1097/MOP.0000000000000191 PMC4535724 25635586

[82] Shah A Coburn CG Watson-Siriboe A Whitley R Shahidzadeh A Gillard ER. Altered cardiovascular reactivity and osmoregulation during hyperosmotic stress in adult rats developmentally exposed to polybrominated diphenyl ethers (PBDEs). Toxicol Appl Pharmacol (2011) 256:103–13. doi: 10.1016/j.taap.2011.07.014 21821059

[83] Rice DC Thompson WD Reeve EA Onos KD Assadollahzadeh M Markowski VP. Behavioral changes in aging but not young mice after neonatal exposure to the polybrominated flame retardant decaBDE. Environ Health Perspect (2009) 117:1903–11. doi: 10.1289/ehp.11814 PMC2799465 20049210

[84] Khalil A Cevik SE Hung S Kolla S Roy MA Suvorov A. Developmental exposure to 2,2′,4,4′-tetrabromodiphenyl ether permanently alters blood-liver balance of lipids in Male mice. Front Endocrinol (2018) 9:548. doi: 10.3389/fendo.2018.00548 PMC6158338 30294300

